# RpoN2- and FliA-regulated *fliTX* is indispensible for flagellar motility and virulence in *Xanthomonas oryzae* pv. *oryzae*

**DOI:** 10.1186/s12866-017-1083-6

**Published:** 2017-08-09

**Authors:** Chao Yu, Huamin Chen, Fang Tian, Fenghuan Yang, Chenyang He

**Affiliations:** State Key Laboratory for Biology of Plant Diseases and Insect Pests, Institute of Plant Protection, Chinese Academy of Agricultural Sciences, Beijing, 100193 China

**Keywords:** *Xanthomonas oryzae* pv. *oryzae*, Flagellar motility, Pathogenicity, Induction of hypersensitive response, T3SS

## Abstract

**Background:**

Bacterial blight of rice caused by *Xanthomonas oryzae* pv. *oryzae* (Xoo) is one of the most important crop diseases in the world. More insights into the mechanistic regulation of bacterial pathogenesis will help us identify novel molecular targets for developing effective disease control strategies. A large flagellar gene cluster is regulated under a three-tiered hierarchy by σ^54^ factor RpoN2 and its activator FleQ, and σ^28^ factor FliA. A hypothetical protein gene *fliTX* is located upstream of *rpoN2*, however, how it is regulated and how it is related to bacterial behaviors remain to be elucidated.

**Results:**

Sequence alignment analysis indicated that FliTX in Xoo is less well conserved compared with FliT proteins in *Escherichia coli*, *Salmonella typhimurium*, and *Pseudomonas fluorescens*. Co-transcription of *fliTX* with a cytosolic chaperone gene *fliS* and an atypical PilZ-domain gene *flgZ* in an operon was up-regulated by RpoN2/FleQ and FliA. Significantly shorter filament length and impaired swimming motility were observed in ∆*fliTX* compared with those in the wildtype strain. ∆*fliTX* also demonstrated reduced disease lesion length and *in planta* growth in rice, attenuated ability of induction of hypersensitive response (HR) in nonhost tobacco, and down-regulation of type III secretion system (T3SS)-related genes. *In trans* expression of *fliTX* gene in ∆*fliTX* restored these phenotypes to near wild-type levels.

**Conclusions:**

This study demonstrates that RpoN2- and FliA-regulated *fliTX* is indispensible for flagellar motility and virulence and provides more insights into mechanistic regulation of T3SS expression in Xoo.

**Electronic supplementary material:**

The online version of this article (doi:10.1186/s12866-017-1083-6) contains supplementary material, which is available to authorized users.

## Background

Bacterial leaf blight caused by *Xanthomonas oryzae* pv. *oryzae* (Xoo) is a major bacterial disease of rice in Asian countries, which can lead to 20%–50% yield loss in rice production [1]. Xoo has been used as a model pathogen to study the molecular mechanism of bacterial pathogenesis in monocotyledonous plants [2, 3]. Now, we have learned that Xoo produces multiple virulence factors, such as exopolysaccharide (EPS), extracellular enzymes, adhesins, and the type III secretion system (T3SS) and its effectors [1, 4, 5]. HrpG and HrpX are the two master regulators to control the expression of *hrp* genes and type III effector genes [6]. Moreover, some other regulators controlling the expression of these virulence factors have also been identified [6–8]. One of the important regulators is alternative sigma factor σ^54^ encoded by *rpoN2* [9, 10]. Deletion of *rpoN2* significantly reduces virulence and flagellar motility, yet how exactly RpoN2 regulates these virulence phenotypes in Xoo remains unknown [10].

The flagellum is the main motor organ in bacteria, which helps bacteria move toward favorable conditions and become infectious [11–13]. The flagellum consists of three parts, the basal body, the hook, and the filament. The regulatory network of flagellar gene transcription is quite complicated and fascinating. In *Escherichia coli* and *Salmonella typhimurium*, over 650 genes involved in flagellum assembly are organized into a hierarchy of three classes [14–16]. The FlhDC encoded by the class I gene *flhDC* is the master regulator and controls the transcription of class II genes [17]. The class II gene products include most of flagellum structural components and alternative sigma factor FliA (σ^28^). FliA regulates the transcription of class III genes, which encode the hook-associated proteins FlgK and FlgL, the anti-σ^28^ factor FlgM, the flagellar cap FliD, the flagellin FliC and other proteins involving in chemosensory signal transduction [18, 19]. The flagellar gene cluster of *Pseudomonas aeruginosa* has a four-tiered hierarchy of transcriptional regulation. Class I genes encode the σ^54^ factor RpoN and σ^54^-dependent transcriptional activator FleQ. Class II genes include the two-component system *fleSR* and the σ^28^ factor *fliA*. The transcription of *fleSR* and *fliA* are regulated by RpoN and FleQ. Class III genes are regulated by FleR and are necessary for completion of the basal-body hook structure. Class IV genes are transcribed by FliA and encode the flagellin and some chemotaxis proteins [20, 21].

FliT is a key chaperone in the flagellar assembly and operation, which interacts with several flagellar proteins, including the filament-cap FliD, the export apparatus components FliI (ATPase), FliJ and FlhA, and the master regulator FlhDC [22–27]. FliT binds to the cognate substrates to not only prevent them from degradation and aggregation in the cytoplasm, but also efficiently transfer them to the export apparatus [28]. The structural analysis has showed that FliT adopts an auto-inhibited conformation, in which both the substrate- and FlhA-binding sites are occluded. Formation of FliT-substrate complex activates its binding to FlhA and thus targeting of the complex to the export gate [29]. In addition, FliT acts as a negative regulator of flagellar regulon and inhibits the binding of FlhDC to the promoter DNA [27, 30]. Interestingly, deletion of *fliT* does not affect the swimming ability in *S*. *typhimurium*, but significantly reduces motility properties in *P. fluorescens* [23, 31]. Moreover, disruption of *fliT* induces the expression of *Salmonella* pathogenicity island 1 (SPI1) genes, implying the potential role of FliT in bacterial virulence [32].

Our previous study has showed that over 60 contiguous flagellar genes forming a large gene cluster in Xoo PXO99^A^ encode proteins with various functions, including structural components, protein export apparatus, regulatory factors, post-translational modification enzymes, and chemotaxis proteins [10]. These genes were tightly regulated under a three-tiered hierarchy by σ^54^ factor RpoN2, and transcriptional activator FleQ, and σ^28^ factor FliA. Interestingly, a hypothetical protein gene *PXO_06168*, named as *fliTX,* has been revealed upstream of *rpoN2* and downstream of *fliS*, which is very similar location of the *fliT* genes in the genome of *S*. *typhimurium* and *P. fluorescens*. However, how *fliTX* is regulated and how it is related to bacterial behaviors, such as flagellar motility and virulence, remain to be elucidated.

In this study, we characterized the regulation and biological functions of *fliTX*. Promoter activities and quantitative real-time polymerase chain reaction (qRT-PCR) assays demonstrated that the transcription of *fliTX* was up-regulated by RpoN2, FleQ and FliA. In frame deletion of *fliTX* led to significant changes in flagellar motility, pathogenicity on rice, hypersensitivity on tobacco, and T3SS-related gene transcription, suggesting that FliTX plays key roles in controlling flagellar motility and virulence in Xoo.

## Results

### Identification, deletion and complementation of *fliTX*

**Fig. 1:**
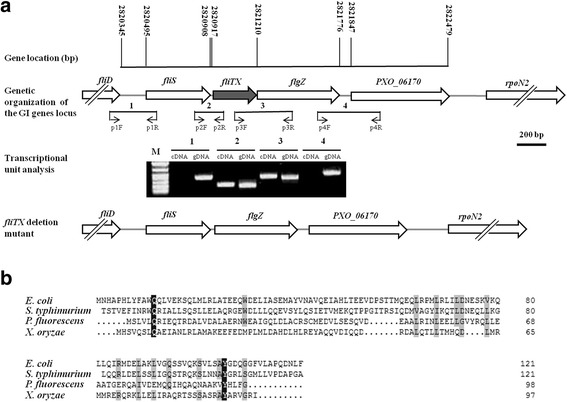
Bioinformatics analysis of *fliTX* in *Xanthomonas oryzae* pv. *oryzae*. **a** Schematic diagram of the region including *fliTX* in the genome of PXO99^A^. Open arrows indicate length, location and orientation of the ORFs. The short lines below the arrows indicate the location and length of RT-PCR products. The lower element shows the RT-PCR analysis of RNA isolated from PXO99^A^. RT-dependent amplification of DNA fragments suggested that the *fliS*, *fliTX* and *flgZ* were transcribed in one operon. The lowest element shows the *fliTX* was in-frame deleted in ∆*fliTX*. **b** Sequence alignment of FliTX was performed using DNAMAN software. The amino acid sequences of FliT were obtained from the National Center for Biotechnology Information (NCBI) website. *E. coli*: *Escherichia coli* strain MG1655; *S*. *typhimurium Salmonella typhimurium* LT2 strain; *P. fluorescens*: *Pseudomonas fluorescens* strain F113; *X*. *oryzae*: *Xanthomonas oryzae* pv. *oryzae* strain PXO99^A^. The amino acid residues highlighted with black means the homology level is 100%

Our previous study showed that there is a flagellar regulon containing over 60 contiguous genes in the genome of Xoo strain PXO99^A^, which are regulated by RpoN2 and FleQ [10]. Upstream of *rpoN2*, there were five genes encoding a filament cap protein FliD (PXO_06166), a cytosolic chaperone FliS (PXO_06167), a hypothetical protein FliTX (PXO_06168), a non-canonical PilZ-domain protein FlgZ (PXO_06169), and a DNA-binding response regulator (PXO_06170) (Fig. 1a). The intergenic distances of neighboring genes are 150 bp, 9 bp, −1 bp, and 71 bp, respectively. Reverse transcription polymerase chain reaction (RT-PCR) analysis was performed to determine whether these five genes form an operon. The fragments containing junctions of *fliS*-*fliTX* and *fliTX*-*flgZ* were obtained using the Xoo cDNA as the template (Fig. 1a), indicating that *fliS*, *fliTX* and *flgZ* are co-transcribed in an operon. Sequence alignment analysis indicated that FliTX was less well conserved compared with FliT proteins in *Escherichia coli*, *Salmonella typhimurium*, and *Pseudomonas fluorescens* (Fig. 1b). To further identify the role of FliTX in Xoo, an in-frame deletion mutant ∆*fliTX* and its complementary strain ∆*fliTX*-C were generated as described in the Materials and Methods. DNA sequencing analysis showed that the corresponding region of *fliTX* was completely deleted in ∆*fliTX*.

### *fliTX* is transcriptionally up-regulated by RpoN2, FleQ and FliA

**Fig. 2:**
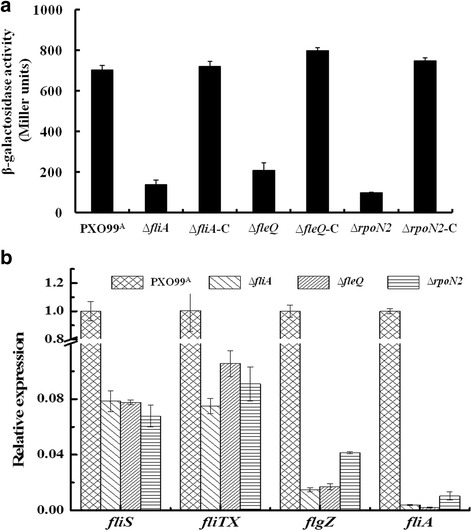
Regulation of *fliTX* transcription in *Xanthomonas oryzae* pv. *oryzae*. **a** β-galactosidase activity assay. Activities of the *fliS* promoter in Xoo strains were detected. The experiments were repeated three times, independently. **b** qRT-PCR analysis of genes in *fliS* operon and *fliA* in Xoo strains. The data represents the relative expression level of genes in PXO99^A^, ∆*fliA*, ∆*fleQ* and ∆*rpoN2*. The error bar represents standard deviations from three biological repeats

To identify whether and how *fliTX* is regulated in Xoo, the promoter activities of the *fliS*-*fliTX*-*FlgZ* operon were examined by measuring β-galactosidase activity of the *fliSp-lacZ* fusion in ∆*rpoN2*, ∆*fleQ*, ∆*fliA*, and the relevant complementary strains. β-galactosidase activity of *fliSp-lacZ* was significantly reduced in ∆*rpoN2*, ∆*fleQ*, and ∆*fliA* compared with that in the wild type, and restored in the relevant complementary strains (Fig. 2a). qRT-PCR analysis showed that transcripts of *fliS*, *fliTX*, and *FlgZ* were dramatically decreased in ∆*rpoN2*, ∆*fleQ*, and ∆*fliA* compared with that in wild type (Fig. 2b), indicating that the transcription of the *fliS*-*fliTX*-*FlgZ* operon was regulated by RpoN2, FleQ, and FliA. Consistent with our previous report [10], the transcription of *fliA* was also significantly decreased in ∆*rpoN2* and ∆*fleQ* (Fig. 2b). These results strongly suggest that RpoN2/FleQ regulate the transcription of the *fliS*-*fliTX*-*FlgZ* operon via FliA in Xoo.

### FliTX is required for flagellar motility and filament production

**Fig. 3:**
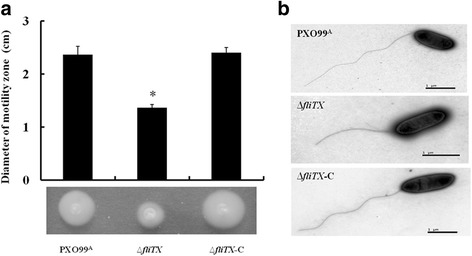
Flagellar motility and filament production of *Xanthomonas oryzae* pv. *oryzae* strains. **a** Assay of swimming motility for PXO99^A^, ∆*fliTX* and ∆*fliTX-C* strains. The swimming zones are recorded after bacterial growth for 4 days on the semisolid plates at 28 °C. Error bars indicate stand deviation. Statistical significance is presented by asterisk (*P* < 0.05, Student’s *t* test). **b** Observation of filament for PXO99^A^, ∆*fliTX* and ∆*fliTX-C* strains using transmission electron microscopy

Since *fliTX* is located within the flagellar regulon, the function of FliTX in flagellar filament assembly and flagellum-dependent motility was investigated. The swimming ability of ∆*fliTX* was detected on the 0.25% agar semisolid plates. Compared with the wild type strain, ∆*fliTX* showed a much smaller swimming zone, and the defect was restored to near wild-type level in the complementary strain containing a plasmid to express the full length of *fliTX in trans* (Fig. 3a). To further determine whether deletion of *fliTX* affected the flagellar biogenesis in Xoo, the single-polar flagellum of various Xoo strains were observed by transmission electron microscope (TEM). The average length of the flagellum on ∆*fliTX* was significantly shorter than that on the wild type, and it was recovered to wild-type level in the complementary strain (Fig. 3b). These results indicate that FliTX is necessary for flagellar biogenesis and motility in Xoo.

### *∆fliTX* shows reduced pathogenicity and bacterial growth in rice

**Fig. 4:**
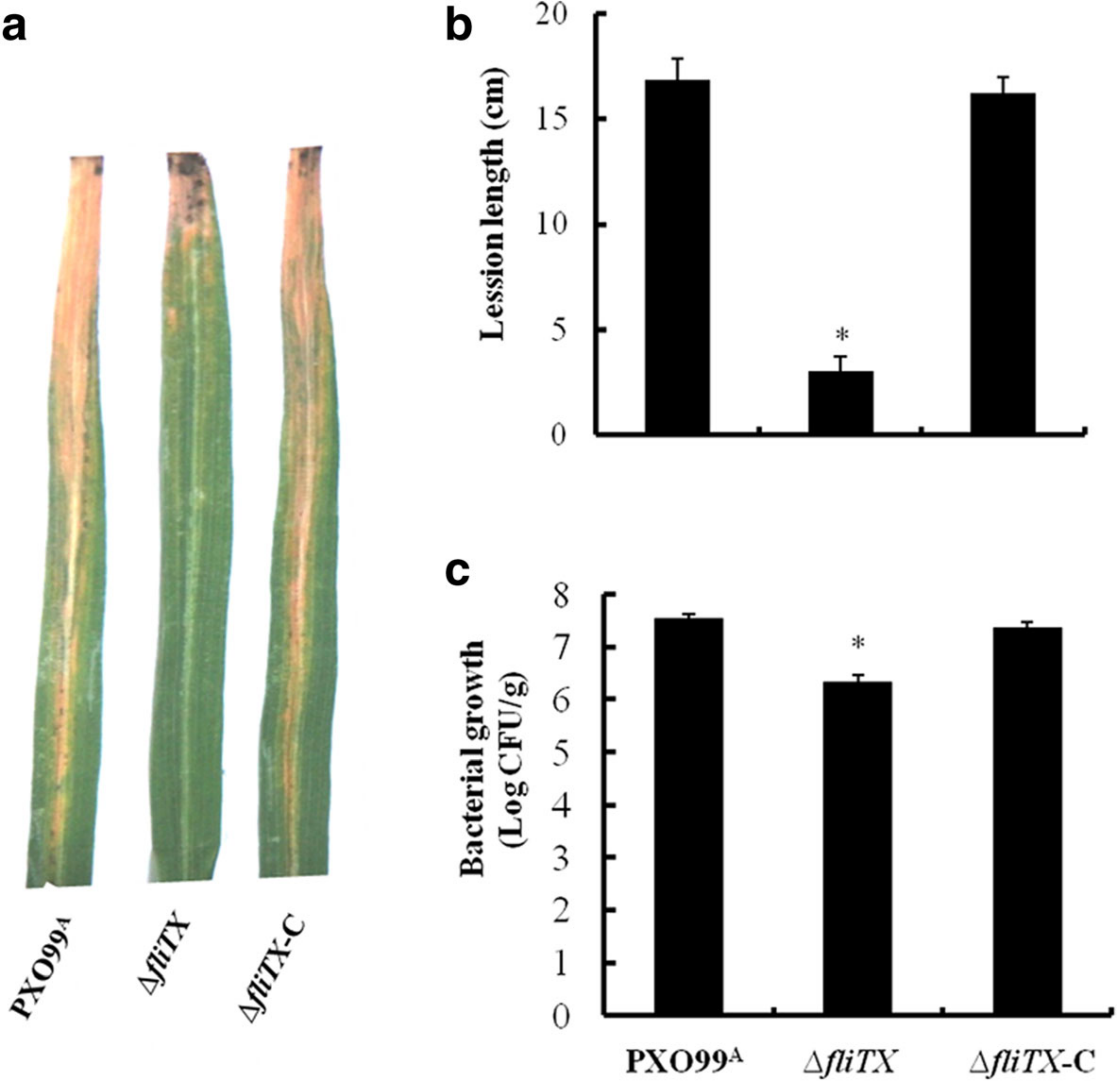
Virulence of *Xanthomonas oryzae* pv. *oryzae* strains in rice. **a** PXO99^A^, ∆*fliTX* and ∆*fliTX-C* strains were inoculated into 6-week-old rice leaves by using the leaf-clipping method. The disease symptoms were observed at 14 days post-inoculation. **b** The lesion lengths were recorded from 10 inoculated leaves for every strain. **c** Bacterial numbers in the top 20 cm of each lesion leaf were scored. Data represent the mean and standard deviations of three independent experiments, and the asterisk above the bars denote statistically significant differences (*P* < 0.05, Student’s *t* test)

To demonstrate the function of *fliTX* in virulence, the pathogenicity of various Xoo strains on susceptible rice cultivar IR24 was tested by the leaf clipping method, and lesion lengths were measured 14 days post inoculation. Compared with the wild type, ∆*fliTX* caused much shorter disease lesion, which were restored in the complementary strain (Fig. 4a and b). Measuring bacterial population in the diseased leaves of rice showed that deletion of *fliTX* significantly led to reduced bacterial population in rice leaves, but complementation with *fliTX* in *trans* restored the bacterial growth *in planta* to near wild-type levels (Fig. 4c). These findings reveal that FliTX is required for virulence of Xoo in rice.

### *∆fliTX* is impaired in the ability to elicit hypersensitive response (HR) in tobacco

**Fig. 5:**
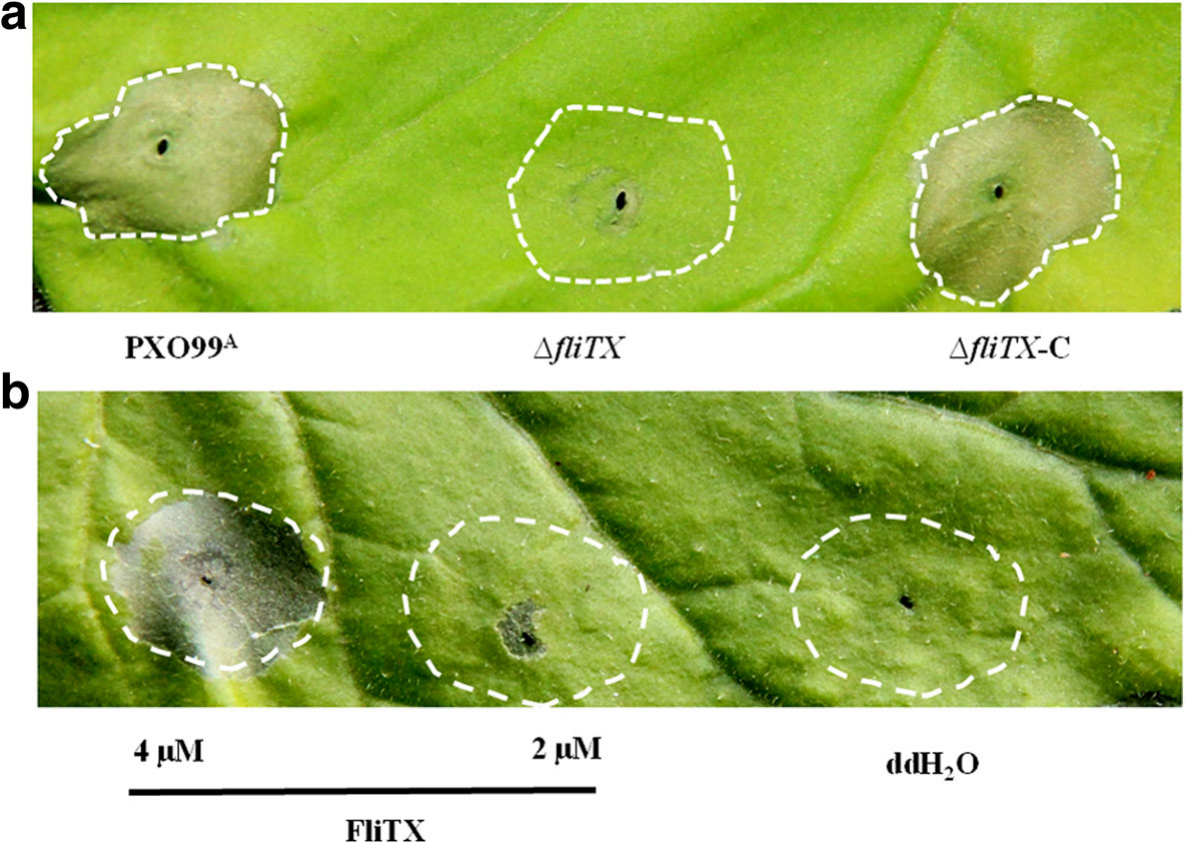
Hypersensitive cell death in tobacco induced by *Xanthomonas oryzae* pv. *oryzae* strains and FliTX protein. Cell suspensions of Xoo strains at OD_600_ of 0.1 (**a**) or recombinant FliTX protein (**b**) were infiltrated onto 6-week-old tobacco leaves. The ddH_2_O was used as control. The HR symptoms were detected and photographed at 24 h post-inoculation. At least four independent experiments were performed with similar results

To unveil the role of FliTX in Xoo when interacting with nonhost plants, HR-inducing ability of Xoo strains on tobacco leaves was tested. Wild type strain induced the typical programmed cell death due to the hypersensitive responses (HR) in the non-host tobacco plants. In contrast, ∆*fliTX* completely lost such an ability to elicit HR, while the complementary strain caused a similar phenotype as the wild type strain (Fig. 5a). We then hypothesized that FliTX protein might be able to elicit HR. To test it, the recombinant protein FliTX-His_6_ was first expressed in *E. coli* strain BL21 and extracted from the soluble fraction by using pre-equilibrated Ni2_resin. SDS-PAGE analysis demonstrated that FliTX-His_6_ was about 14 KDa in size (Additional file 1: Figure S1). Then, the purified protein was infiltrated into tobacco leaves at two different concentrations. HR in tobacco was strongly induced when FliTX-His_6_ was applied at the concentration of 4 μM, while no HR was observed when the concentration was reduced to 2 μM (Fig. 5b). The negative control of sterilized double-distilled water (ddH_2_O) did not cause HR either. These observations demonstrate that FliTX protein plays an important role in Xoo to elicit HR in nonhost tobacco.

### *∆fliTX* was attenuated in T3SS-related gene expression

**Fig. 6:**
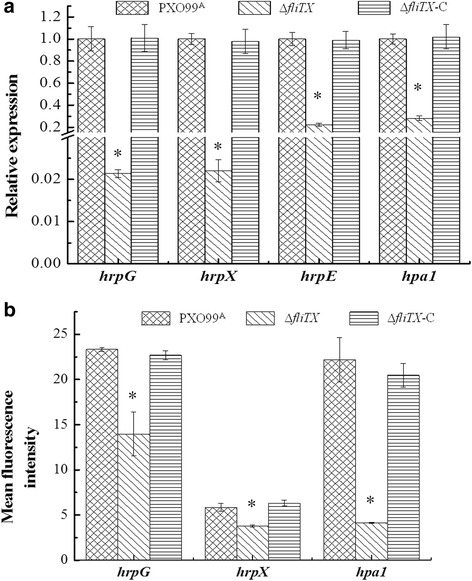
Transcription of T3SS-related genes in *Xanthomonas oryzae* pv. *oryzae* strains. **a** The relative expression of T3SS-related genes was detected by qRT-PCR in PXO99^A^, ∆*fliTX* and ∆*fliTX-C* strains. Fold changes of each gene was calculated using the 2^-∆∆Ct^ method. **b** Promoter activity of *hrpG*, *hrpX* and *hpa1* in PXO99^A^, ∆*fliTX* and ∆*fliTX-C* strains. The promoter of *hrpG*, *hrpX* and *hpa1* were ligated to pPROBE-AT, a broad-host-range vector carrying a promoter-less *gfp* gene, resulting in plasmids pPhrpG, pPhrpX and pPhpa1, respectively. These plasmids, pPhrpG, pPhrpX and pPhpa1, were transferred to *fliTX* deletion mutant, complementary strain and wildtype strains by electroporation. Green fluorescent protein mean fluorescence intensity was determined for gated populations of bacterial cells by flow cytometry. Error bars represent standard deviations from three biological repeats, and asterisk indicates *P* < 0.05 by Student’s *t* test

The HR-inducing ability on nonhost and pathogenicity on host (Hrp) is closely related to T3SS in pathogenic bacteria [33, 34]. To understand the function of FliTX in T3SS in Xoo, transcripts of T3SS-related *hrp* genes were measured through qRT-PCR analysis. Compared with that in the wild type, transcription levels of *hrpG*, *hrpX*, *hrpE* and *hpa1* were significantly decreased in ∆*fliTX*, and restored near to wild-type level in the complementary strain (Fig. 6a). Moreover, promoter activities of *hrpG*, *hrpX* and *hpa1* revealed through flow cytometry analysis were dramatically reduced in ∆*fliTX* compared with that of the wild type. All promoter activities were restored to wild-type levels in the complementary strain (Fig. 6b). Since HrpG controls the transcript of other *hrp* genes via regulating *hrpX* expression in *Xanthomonas* [35], these results suggest that FliTX positively regulates the expression of T3SS in Xoo through the master regulator HrpG.

## Discussion

In the current study, we identified a novel flagellar gene *fliTX*, determined its expression patterns, and assessed its functions in motility and virulence on rice through bioinformatics and genetic analysis. We demonstrated that the transcription of *fliTX* was positively regulated by RpoN2/FleQ and FliA. We also revealed that *fliTX* was indispensible for bacterial phenotypes, including flagellar motility, pathogenicity in rice, induction of HR in tobacco, and T3SS-related gene expression. Therefore, our identification of FliTX provides more insights into mechanistic regulation of motility and virulence in Xoo.

An over 60 contiguous gene containing cluster has been shown to putatively encode flagellar proteins with various functions, including structural components, protein export apparatus, regulatory factors, post-translational modification enzymes/proteins, and chemotaxis proteins in Xoo [9, 10]. The flagellar assembly and operation are tightly regulated under a three-tiered hierarchy by RpoN2/FleQ and FliA [10]. Based on the gene location and transcription feature, we found that *fliTX* was transcribed in the *fliS*-*fliTX*-*flgZ* operon regulated by RpoN2/FleQ and FliA (Figs.1 and 2). This is quite different from the *fliD*-*fliS*-*fliT* operon in other pathogenic bacteria including *E. coli* and *S*. *typhimurium* [36, 37]. The significantly reduced transcripts of *fliA* in ∆*rpoN2* and ∆*fleQ* (Fig. 2b) suggest that regulation of transcription of the *fliS*-*fliTX*-*flgZ* operon by RpoN2/FleQ might be through FliA under a three-tiered hierarchy.

The varied functions of FliT in flagellar motility have been shown in several pathogenic bacteria. For example, FliT has been described as the filament-capping protein FliD substrate-specific chaperone in *S*. *typhimurium* [24]. Deletion of *fliD* inhibited the assembly of flagellin molecules onto the hooks, resulting in failure to filament biogenesis [37, 38]. However, no difference in swimming ability was observed between wildtype and the mutant [23]. In contrast, ∆*fliT* showed normal flagellar filaments but attenuated swimming motility in *P. fluorescens* F113 [31], suggesting that FliT might not act as a FliD chaperone. Our current observation that FliT was less conserved in the strains of *E. coli*, *S*. *typhimurium*, *P. fluorescens* and Xoo (Fig. 1) implicates that FliT may function differentially in flagellar motility in various bacteria. In this study, in frame deletion of *fliTX* led to significantly abnormal filaments and reduced swimming motility (Fig. 3), demonstrating that FliTX plays important roles in filament assembly and motility in Xoo. Further experiments are required to determine whether FliTX functions as a FliD chaperone to affect the flagellar motility in Xoo.

The role of FliT in bacterial virulence has only been reported in *S*. *typhimurium*, in which the transcription of T3SS-containning SPI1 was induced upon disruption of *fliT*, and the repressive effect of *fliT* on SPI1 genes was completely abolished in ∆*flhDC* [32], indicating that FliT negatively regulates the virulence and related gene expression in the FlhDC-dependent manner in *Salmonella*. In this study, we demonstrated that in frame deletion of *fliTX* resulted in dramatically reduced lesion length and bacterial growth in rice (Fig. 4), and impaired HR-inducing ability in tobacco (Fig. 5a). It has been known that the T3SS plays critical roles in conferring pathogenicity on the host and triggering the HR on nonhost plants by delivering effector proteins into plant cells [33, 34]. Meanwhile, in the current study, we showed that the expression of two T3SS regulator genes, *hrpG and hrpX*, were attenuated in the ∆*fliTX* mutant (Fig. 6). Thus, this study provides for the first time the experimental evidence that FliTX functions to promote the bacterial virulence via regulating T3SS gene expression in Xoo. Based on our previous demonstration that RpoN2 positively regulates the virulence on rice through an unknown manner [10], and the current observation that *fliTX* is up-regulated by RpoN2 and required for the virulence in Xoo, it is reasonable to speculate that FliTX works in the RpoN2-dependent pathway to promote the bacterial pathogenesis in rice. More investigations are needed to confirm this hypothesis and further understand the regulatory mechanism of virulence by FliTX in Xoo.

For the assembly of bacterial flagellum for motility, the flagellar type III export apparatus utilizes both ATP and proton motive force to cross the cytoplasmic membrane and export flagellar proteins from the cytoplasm to the cell membrane [28]. FliT acts as the specific chaperone of the filament-capping protein FliD that is protected from degradation and aggregation in the cytoplasm and efficiently transferred to the distal end of the flagellar structure [26]. However, it remains mysterious whether and how the FliT protein is secreted. In addition, we showed that the recombinant FliTX protein induced HR in monhost tobacco leaves (Fig. 5b), implicating a potential role of FliTX in inducing plant defense responses. Therefore, it is required to further demonstrate whether and how FliTX is secreted into the plant cells during the induction of HR.

## Conclusions

The *fliTX* gene is transcriptionally up-regulated by RpoN2/FleQ and FliA, and necessary for flagellar assembly and motility in Xoo. Deletion of *fliTX* led to significantly reduced virulence in rice, attenuated ability of induction of HR in tobacco and decreased *hrp* gene expression. RpoN2/FleQ- and FliA-regulated FliTX controls the bacterial pathogenesis via T3SS regulation with the unknown manner(s).

## Methods

### Bacterial strains and culture conditions

*Xanthomonas oryzae* pv. *oryzae* wildtype strain PXO99^A^ and derived mutants were grown in peptone sucrose agar (PSA) medium [39] or M210 liquid medium [40] at 28 °C, *Escherichia coli* DH5α and BL21 strains were grown in Luria-Bertani (LB) medium at 37 °C. The antibiotics used were ampicillin (Ap), gentamicin (Gm), kanamycin (Km) and spectinomycin (Sp) at concentrations of 100, 50, 50, and 50 μg/mL, respectively. The bacterial strains and plasmids used in this study are listed in Table 1.

**Table 1:** Bacterial strains and plasmids used in this study

Strain or plasmid	Relevant characteristics^a^	Source or Reference
*Escherichia coli*		
DH5α	supE44 ΔlacU169(Φ80lacZΔM15) hsdR17 recA1 endA1 gyrA96 thi-1 relA1	[49]
BL21	For protein expression	Novagen
*Xanthomonas oryzae* pv. *oryzae*		
PXO99^A^	Wildtype strain, Philippine race 6	Lab collection
∆*fliTX*	*fliTX* gene deletion mutant derived from PXO99^A^	This study
∆*fliTX-C*	Complementary strain of ∆*fliTX,* Ap^r^	This study
∆*rpoN2*	*rpoN2* gene deletion mutant derived from PXO99^A^, Gm^r^	Our lab
∆*rpoN2-C*	Complementary strain of ∆*rpoN2,* Ap^r^	Our lab
∆*fleQ*	*fleQ* gene deletion mutant derived from PXO99^A^, Gm^r^	Our lab
∆*fleQ-C*	Complementary strain of ∆*fleQ,* Ap^r^	Our lab
∆*fliA*	*fliA* gene deletion mutant derived from PXO99^A^, Gm^r^	Our lab
∆*fliA-C*	Complementary strain of ∆*fliA,* Ap^r^	Our lab
Plasmid		
pMD18-T	Cloning vector, Ap^r^	TaKaRa, Tokyo
pKMS1	Suicidal vector carrying *sacB* gene for non-marker mutagenesis, Km^r^	[45]
pBBR1MCS-4	Broad-host range expression vector, Ap^r^	[50]
pHT304BZ	Promoterless *lacZ* vector, Ap^r^	[41]
pHTpS	pHT304BZ derivative carrying the promoter region of *fliS*, Ap^r^	This study
pHM1	Broad-host range expression vector, Sp^r^	[42]
pH-*fliSp*-*lacZ*	pHM1 derivative carrying the promoter region of *fliS* and promoterless *lacZ*, Sp^r^	This study
pET-28a	Expression vector to generate a N-terminal His_6_ tag, Km^r^	Haigene
pET-*fliTX*	pET-28a derivative carrying *fliTX*, Km^r^	This study
pPROBE-AT	broad-host-range vector carrying a promoter-less *gfp* gene, Ap^r^	[47]
pPhrpG	pPROBE-AT derivative carrying the promoter region of *hrpG* and promoterless *gfp*, Ap^r^	This study
pPhrpX	pPROBE-AT derivative carrying the promoter region of *hrpX* and promoterless *gfp*, Ap^r^	This study
pPhpa1	pPROBE-AT derivative carrying the promoter region of *hpa1* and promoterless *gfp*, Ap^r^	Our lab

^a^Ap^r^, Km^r^, Sp^r^, and Gm^r^ indicate resistant to ampicillin, kanamycin, spectinomycin and gentamicin, respectively

### Bioinformatics analysis of *fliTX*

The domain organization of FliTX was analyzed using online software available at the SMART Website (http://smart.embl-heidelberg.de/). The amino acid sequences of FliTX were obtained from the National Center for Biotechnology Information (NCBI) website. BLASTP was using for searching the homology in *Xanthomonas* species. Relevant sequence alignment was performed using the DNAMAN software (Lynnon Biosoft, San Ramon, USA).

### Generation of *lacZ* fusion and assay for β-galactosidase activity

The promoter region (−309 to −1) of *fliS* was amplified from PXO99^A^ genomic DNA using specific primers fliSpF/R (Additional file 2: Table S1), and ligated into the *Hind*III and *BamH*I sites of the pHT304BZ vector containing a promoterless *lacZ* reporter gene [41]. Recombinant pHTpS was verified by DNA sequencing (Beijing Genomics Institute, Beijing) and treated with HindIII and KpnI, and the fragment containing *fliS* promoter region and the promoterless *lacZ* reporter gene was obtained and then cloned into plasmid pHM1 [42]. Finally, the recombinant plasmid pH-*fliSp*-*lacZ* was generated and introduced into PXO99^A^ and derived mutants. The resultant strains contained pH-*fliSp*-*lacZ* were selected by resistance to spectinomycin and verified by polymerase chain reaction (PCR). For β-galactosidase assay, these Xoo strains were cultured in M210 liquid medium at 28 °C and 200 rpm, and till an optical density (OD_600_) of 1.0, cells were collected by centrifugation at 12,000 g. The β-galactosidase activity was determined using the β-Galactosidase Enzyme Assay System (Promega, Wisconsin, USA). The experiments were repeated three times, independently.

### RNA isolation and qRT-PCR analysis

RNA isolation and qRT-PCR analysis were performed as described previously with some modifications [8]. Briefly, Xoo strains were grown in M210 liquid medium at 28 °C till OD_600_ of 0.8, and harvested by centrifugation at 12,000 g for analysis of gene expression. For T3SS-related gene assays, the harvested bacterial cells were sub-cultured in XOM2 medium [43] overnight at 28 °C and collected again. Total RNA was extracted with RNAprep pure Cell/Bacteria Kit (Tiangen, Beijing, China) and treated with DNase and cDNA was synthesized from total RNA using the FastQuant RT Super Mix (Tiangen, Beijing, China). RT-qPCR was performed using Quant qRT-PCR kit (Tiangen, Beijing, China) in Applied Biosystem’s 7500 (Applied Biosystems, Foster City, CA, USA) with gene specific primers, and *gyrB* was used as a reference gene (Additional file 2: Table S1). The relative expression ratio was calculated using 2^–∆∆Ct^ method [44]. These experiments were performed in three biological replicates and triplicate PCR.

### Protein expression and purification

The FliTX expression and purification were performed as described previously [8]. Briefly, the coding region for *fliTX* was amplified by PCR with primers TXF/R (Additional file 2: Table S1) and ligated to the middle vector pMD18-T for verification by DNA sequencing. Then the *fliTX* fragment was digested from verified pMD18-T with corresponding restriction enzymes and ligated to pET28a, resulting in pET-*fliTX*. The recombinant plasmid was transformed into *E. coli* BL21 strain for protein expression. For protein purification, the BL21 strain carrying pET-*fliTX* was cultured in LB liquid medium at 37 °C to OD_600_ of 0.6, and isopropyl-thio-galactopyranoside at a final concentration of 0.1 mM was added to induce *fliTX* expression. After 6 h cultured, the BL21 cells were collected by centrifugation and re-suspended in 0.1× PBS. The crude cell extracts were obtained by sonication and centrifuged at 12,000 g for 10 min in 4 °C. The supernatant containing the soluble proteins was mixed with pre-equilibrated Ni2_resin (GE Healthcare, Piscataway, NJ, USA) for 1 h at 4 °C. Finally, the FliTX protein combined to Ni was eluted with elution buffer (20 mM Tris-HCl, 350 mM NaCl, 0.5 mM EDTA, 10% glycerol, 5 mM MgCl_2_ and 100 mM imidazole, pH 8.0) and dialyzed with 0.1× PBS. The purified FliTX was detected by sodium dodecyl sulfate polyacrylamide gel electrophoresis (SDS-PAGE) and adjusted to 10 μM with 0.1 × PBS for the next experiments.

### Gene deletion and complementation

An in-frame gene deletion mutant Δ*fliTX* derived from PXO99^A^ was constructed through homologous recombination by using the suicide vector pKMS1 [45]. About 900 bp upstream and 800 bp downstream fragments of the *fliTX* gene were amplified by PCR from Xoo genomic DNA using primers fliTXLF/R and fliTXRF/R, respectively. The PCR products were first cloned into the middle vector pMD18-T (Takara, Dalian, China) and verified by sequencing. Then the upstream and downstream fragments of *fliTX* were digested with corresponding restriction enzymes from the middle vectors and ligated into pKMS1. The final vector pKMS1 containing upstream and downstream fragments of *fliTX* was introduced into Xoo by electroporation. The transformants were first selected on NAN medium (1% tryptone, 0.1% yeast extract, 0.3% peptone and 1.5% agar) with Km, and after continuous transfer cultured in NBN medium (1% tryptone, 0.1% yeast extract and 0.3% peptone) at least five times. Finally, the Δ*fliTX* mutant was selected on NAS medium (1% tryptone, 0.1% yeast extract, 0.3% peptone, 10% sucrose and 1.5% agar) and further confirmed by PCR analysis. For complementation strain construction, the full length of *fliTX* was amplified by PCR with primers fliTXF/R and inserted into vector pMD18-T. After verifying by sequencing, *fliTX* was digested from pMD18-T and ligated into pBBR1MCS-4. The final vector pBBR1MCS-4 containing *fliTX* was electroporated into Δ*fliTX* and confirmed by PCR analysis, resulting in the Δ*fliTX* complementary strain (Δ*fliTX*-C). The primers are listed in Additional file 2: Table S1.

### Motility assay and electron microscopy visualization of filament

For motility assay, bacterial strains were cultured in M210 liquid medium till reached OD_600_ of 1.0 and harvested by centrifugation at 12,000 g for 5 min. Cells were re-suspended in equal volume of ddH_2_O. Two microliters of bacterial suspension were spotted onto semisolid plates (0.03% peptone, 0.03% yeast extract and 0.25% agar) and incubated at 28 °C. The diameters of the swimming zone were recorded after 4 days. The experiments were repeated three times with five replicates for each time. The TEM assay was performed as described previously [46]. Briefly, bacterial strains were grown on PSA plates at 28 °C for 48 h, and cells were collected and re-suspended with ddH_2_O, then one drop of suspension was deposited onto grids coated with Formvar (Standard Technology, Ormond Beach, FL, USA). The grids with bacteria were stained with 2% uranyl acetate for 30 s, and air drying for 10 min. The bacterial flagella were observed by TEM using Hitachi H-7500 electron microscope.

### Pathogenicity test

As described above, bacterial strains were cultured in M210 liquid medium at 28 °C and 200 rpm till reached OD_600_ of 1.0, and collected by centrifugation at 12,000 g for 5 min, and re-suspended with equal volume of ddH_2_O. For the disease lesion length assay, bacterial cells were inoculated into leaves of 8-week-old rice (*Oryza sativa ssp. indica*) cultivar IR24 using the leaf-clipping method [8], and the lesion length was measured at 14 days post-inoculation. For the bacterial population assay, the top 20 cm of inoculated rice leaves were collected and weighted, then ground into 1 mL of ddH_2_O. The ground mixtures were optional diluted and spread onto the PSA plates. The bacterial colonies were counted after cultured in incubator with 28 °C for 72 h. At least 10 leaves were inoculated for each strain, and the experiments were repeated three times, independently.

### Assay for induction of HR in tobacco

Xoo strains were grown in M210 liquid medium at 28 °C to OD_600_ of 1.0, and collected by centrifugation at 7000 g for 10 min. The cells were re-suspended with ddH_2_O, and adjusted to OD_600_ of 0.1. Then these bacterial cells or purified FliTX protein were inoculated into leaves of 6-week-old tobacco (*Nicotiana benthamiana*) using a needleless syringe. The HR symptoms were detected and photographed at 24 h post-inoculation. The experiments were repeated three times, independently.

### Flow cytometry detection

The plasmid pPhpa1 containing the *hpa1* promoter region and a promoterless *gfp* gene was constructed in our previous studies [40]. Here two near 200-bp fragments containing the promoter region of *hrpG* or *hrpX* were PCR amplified using the primers hrpGpF/R or hrpXpF/R (Additional file 2: Table S1), and ligated to pPROBE-AT, a broad-host-range vector carrying a promoter-less *gfp* gene [47], resulting in plasmids pPhrpG and pPhrpX, respectively. These plasmids, pPhpa1, pPhrpG and pPhrpX, were transferred to *fliTX* deletion mutant, complementary strain and wildtype strains by electroporation. The transformed strains were cultured in M210 liquid medium to OD_600_ of 1.0 and transferred to XOM2 medium for 12 h at 28 °C. The cells were collected by centrifugation at 12,000 g for 5 min and re-suspended with 0.1× PBS. The promoter activities of *hpa1*, *hrpG* and *hrpX* were detected using a FACS-Caliber flow cytometer (BD Bioscience, CA, USA) as previously described [48]. Xoo wildtype carrying a promoterless pBROBE-AT was used as a negative control. The experiments were repeated three times, independently.

### Data analysis

The values of β-galactosidase activity, gene expression level, motility zone, disease lesion length and bacterial population were presented as means ± SD (standard deviations). Student’s *t* test was performed with statistical significance set at the 0.05 confidence level.

## Additional files

Additional file 1: Figure S1. Coomassie blue staining of the FliTX protein expressed and extracted from *E. coli* strain BL21. M: Molecular marker; 1: FliTX in the soluble fraction; 2: purified FliTX; 3: FliTX in the insoluble fraction. (TIFF 424 kb)

Additional file 2: Table S1. The primers used in this study. (DOCX 17 kb)

## Abbreviations

Ap: Ampicillin
ddH_2_O: Sterilized double-distilled water
EPS: Exopolysaccharide
Gm: Gentamicin
HR: Hypersensitive response
Km: Kanamycin
LB: Luria-Bertani
NCBI: National Center for Biotechnology Information
OD_600_: Optical density
PCR: Polymerase chain reaction
PSA: Peptone sucrose agar
qRT-PCR: Quantitative real-time polymerase chain reaction
RT-PCR: Reverse transcription polymerase chain reaction
SD: Standard deviations
SDS-PAGE: Sodium dodecyl sulfate polyacrylamide gel electrophoresis
Sp: Spectinomycin
SPI1: *Salmonella* pathogenicity island 1
T3SS: Type III secretion system
TEM: Transmission electron microscope
Xoo: *Xanthomonas oryzae* pv. *oryzae*
