# Supplementary figures and images for: RpoN2- and FliA-regulated fliTX is indispensible for flagellar motility and virulence in Xanthomonas oryzae pv. oryzae
Source: BMC Microbiol. 2017 Aug 9;17:171. doi: 10.1186/s12866-017-1083-6 (PMC5550985; doi:10.1186/s12866-017-1083-6)

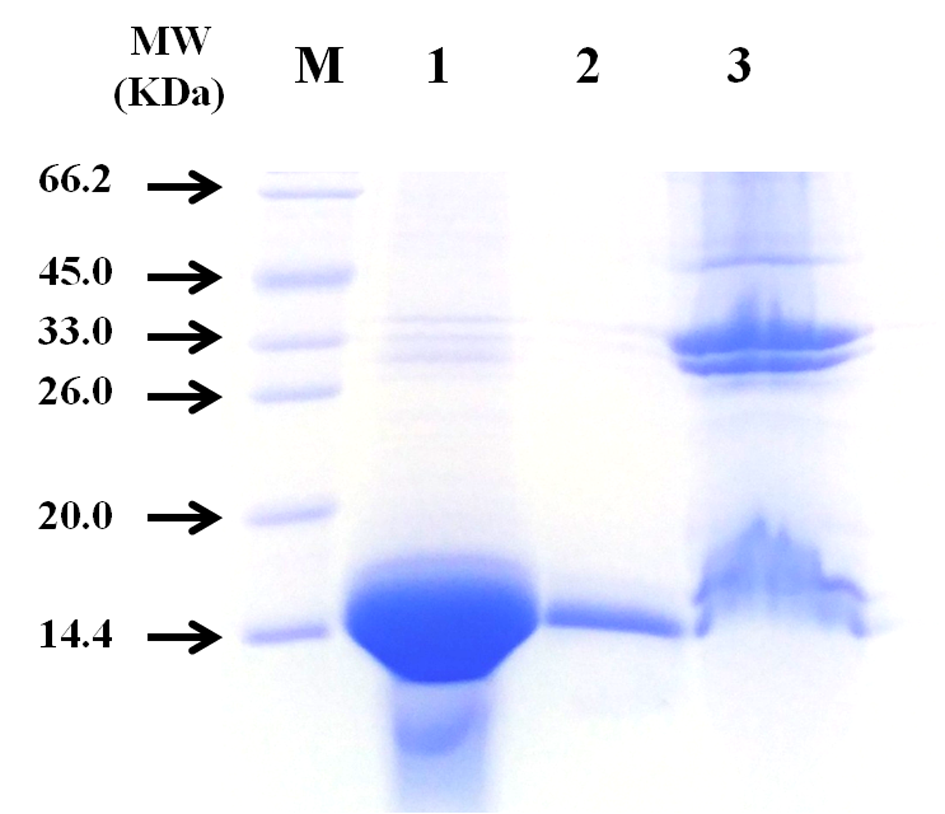

Supplement: Supplementary file 1 — Coomassie blue staining of the FliTX protein expressed and extracted from E. coli strain BL21. M: Molecular marker; 1: FliTX in the soluble fraction; 2: purified FliTX; 3: FliTX in the insoluble fraction. (TIFF 424 kb) [file 12866_2017_1083_MOESM1_ESM.tif]
