# Supplementary material for: RpoN2- and FliA-regulated fliTX is indispensible for flagellar motility and virulence in Xanthomonas oryzae pv. oryzae
Source: BMC Microbiol. 2017 Aug 9;17:171. doi: 10.1186/s12866-017-1083-6 (PMC5550985; doi:10.1186/s12866-017-1083-6)
Supplement: Supplementary file 2 — The primers used in this study. (DOCX 17 kb) [file 12866_2017_1083_MOESM2_ESM.docx]

**Table S1. The primers used in this study**

| **Primer** | **Sequences( 5′- 3′ )** | **use** |
| --- | --- | --- |
| p1F | AAGCAGCAGTTACGACGCCACCACCAA | Transcriptional unit detection |
| p1R | CAAATCCAGCACTTCTGTCAACGCAGTCTCAT | Transcriptional unit detection |
| p2F | ACAAGACCATCAAGGGCTACGAATC | Transcriptional unit detection |
| p2R | CTGAGCAAATCCAGCACTTCTGTCA | Transcriptional unit detection |
| p3F | TAACCTCTCGGCCCTGTACGACTAC | Transcriptional unit detection |
| p3R | CGGTCCTGTTGGATATCCACCTGCT | Transcriptional unit detection |
| p4F | CGAAGACTTGCGTAGCGAGGAA | Transcriptional unit detection |
| p4R | GTCGTGCATCGACATCATCACCACA | Transcriptional unit detection |
| fliTX LF | CGGGATCCCGGCAGCGTCACCGAACTCA | Gene deletion |
| fliTX LR | CCAAGCTTGCTATGCATGACAGGACTCACGAAA | Gene deletion |
| fliTX RF | CCAAGCTTATGAGCACGCTCGGCACACTCG | Gene deletion |
| fliTX RR | GCGTCGACCGGCGATGCAAACGAAACAGG | Gene deletion |
| fliTXF | CCAAGCTTCGCCGTTTCGTGAGTCCTGTC | Gene cloning |
| fliTXR | GCTCTAGATCAGTGATGGTGATGGTGATGGATCCGTCCGACCCGTGCG | Gene cloning |
| fliSpF | CCAAGCTTAACAAGACCATCAAGGGCTAC | *fliS* promoter cloning |
| fliSpR | CGGGATCCGGATGACTCCTCAATGGGCTG | *fliS* promoter cloning |
| fliTXpF | TGTGTTTGGCAAGGACAGCAAG | *fliTX* promoter cloning |
| fliTXpR | GACGATTGGAACCGTACATGG | *fliTX* promoter cloning |
| fliSqF | GCCAACCTGCACAACGATG | qRT-PCR |
| fliSqR | AAGGCCGCGTTGTTCGAG | qRT-PCR |
| fliTXqF | GCAAAGCCTTCAAGCAGAAATCG | qRT-PCR |
| fliTXqR | TCGTGGGCGTCCAGCATCAG | qRT-PCR |
| PXO_06169qF | GCTGTTTGCCGACACGCTTAGTT | qRT-PCR |
| PXO_06169qR | GCGGTCTTCGCTGGTTTCCTC | qRT-PCR |
| fliAqF | CCCAACCCGCAGCAGATGA | qRT-PCR |
| fliAqR | CTGGCCGTGGATCTGGCAG | qRT-PCR |
| hrpGF | TGTCCACCTGATGAACGACCCT | qRT-PCR |
| hrpGR | GGCGAATGCCGCAACGAA | qRT-PCR |
| hrpXF | AGGCACTGACCCACTTTC | qRT-PCR |
| hrpXR | ATCGGAAGCACCACTCTC | qRT-PCR |
| hrpEF | CGTTGTCGCCCGCCTT | qRT-PCR |
| hrpER | GGTTCGTTGCTCGGCG | qRT-PCR |
| hpa1F | AAGCCAGGACACAACGTTCG | qRT-PCR |
| hpa1R | GAAGCAGGGCCGAGATGAG | qRT-PCR |
| gyrBqF | GCGAGCACAATGGCATT | qRT-PCR |
| gyrBqR | CCATCCTTCTGCGGGATGT | qRT-PCR |
| TXF | CGGGATCCGTGCAAAGCCTTCAAGCAGAAATCG | Gene cloning |
| TXR | CCAAGCTTTCAGATCCGTCCGACCCGTGCGT | Gene cloning |
| hrpGpF | CCCAAGCTTTAGTCGGACAACGTCTGCGA | *hrpG* promoter cloning |
| hrpGpR | CGGAATTCCAGGTGGCCATCCCGTG | *hrpG* promoter cloning |
| hrpXpF | CGGAATTCCCTGCTCGTATAGGTAGGAAGAC | *hrpX* promoter cloning |
| hrpXpR | CCCAAGCTTCACGCATTCTGGAATACATCG | *hrpX* promoter cloning |
